# Supplementary material for: Evidence for normal novel object recognition abilities in developmental prosopagnosia
Source: R Soc Open Sci. 2020 Sep 23;7(9):200988. doi: 10.1098/rsos.200988 (PMC7540787; doi:10.1098/rsos.200988)

Lab-tested participants plotted against large normative sample

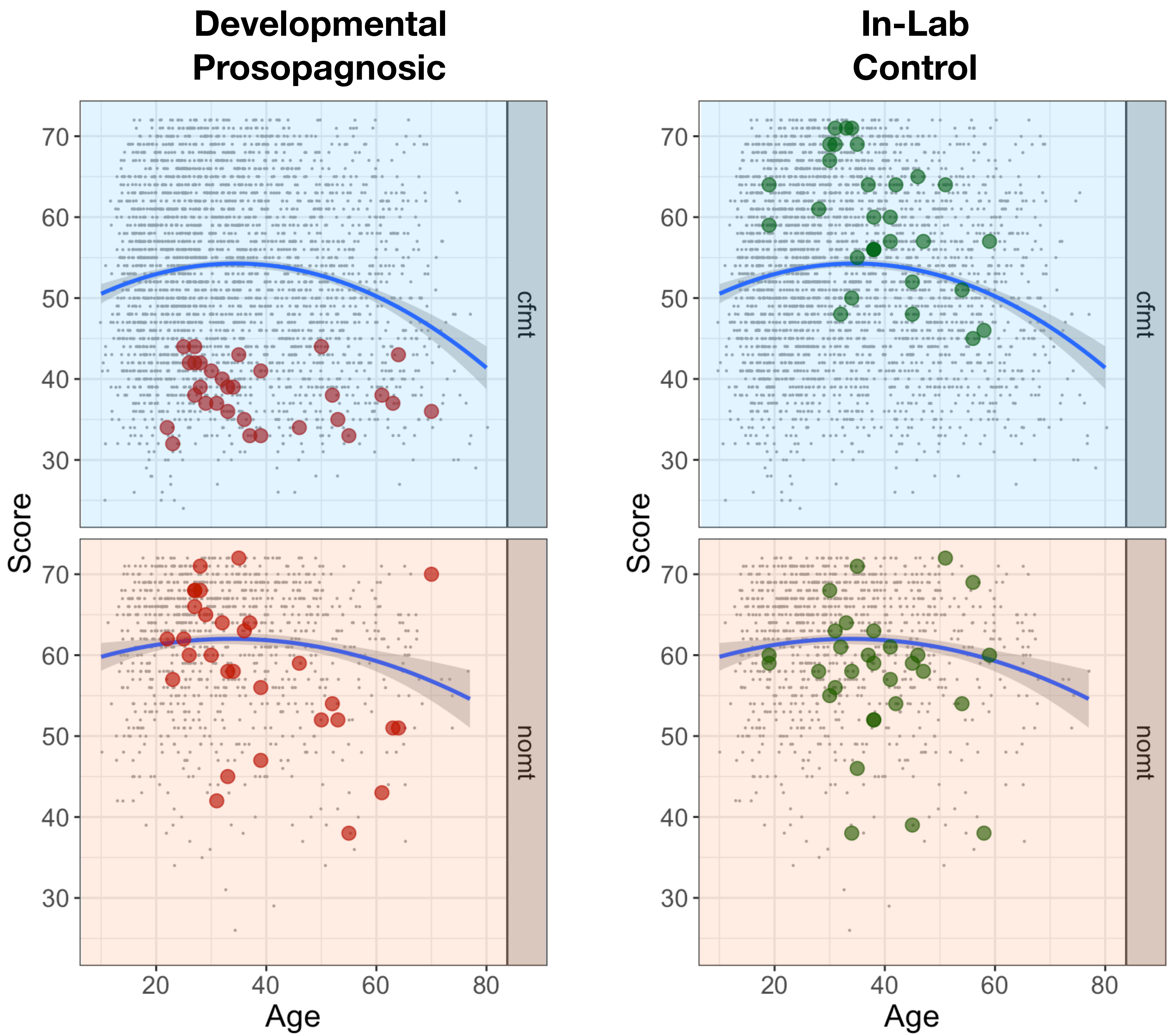

Lab-tested participants plotted against large normative sample

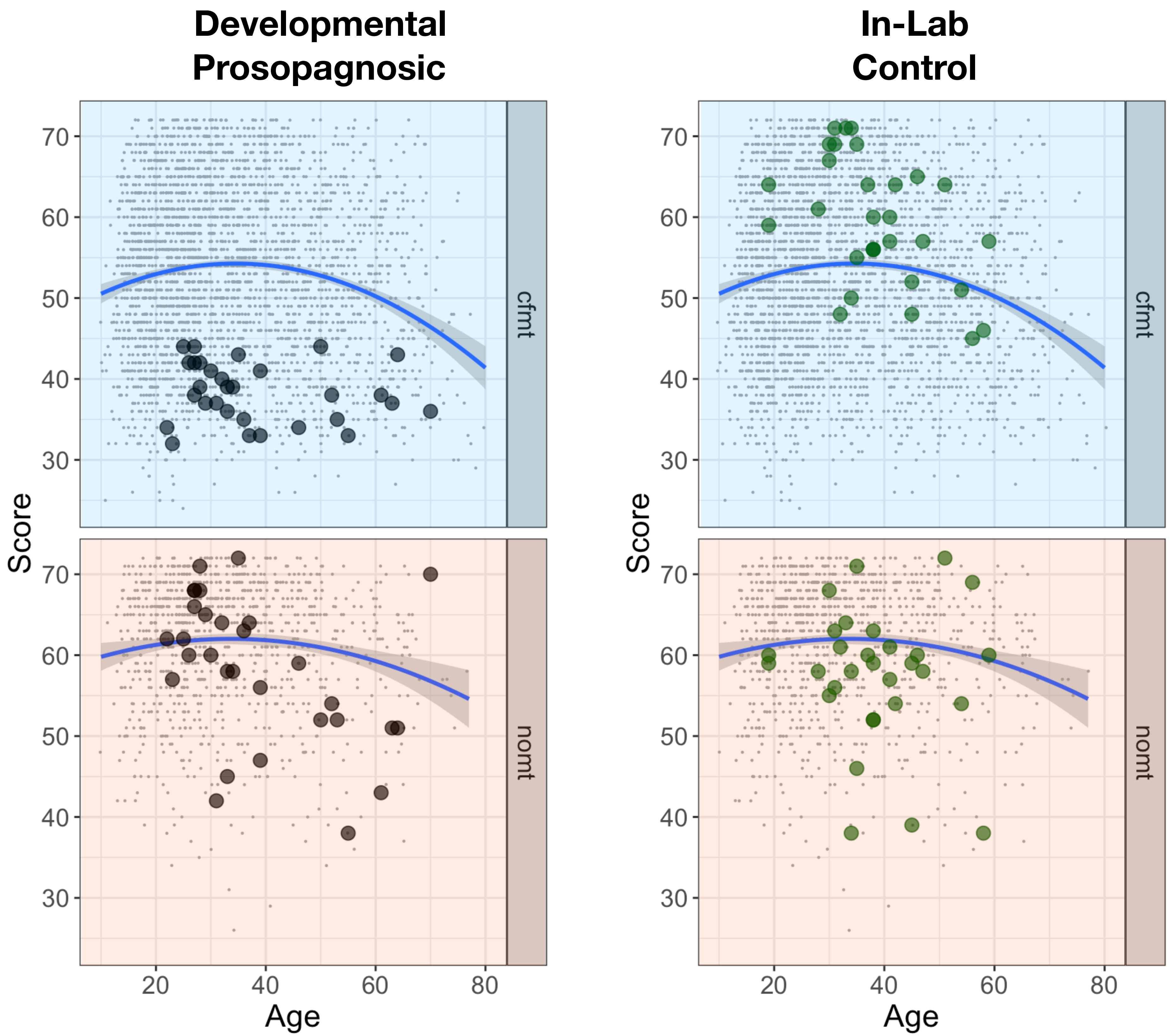

Supplement: Lab-tested participants plotted against large normative sample [file rsos200988supp4.pdf]
