# Supplementary material for: Evidence for normal novel object recognition abilities in developmental prosopagnosia
Source: R Soc Open Sci. 2020 Sep 23;7(9):200988. doi: 10.1098/rsos.200988 (PMC7540787; doi:10.1098/rsos.200988)
Supplement: DP CFMT and NOMT scores [file rsos200988supp5.docx]

**Supplementary Table 1.** DPs’ Raw Data: Demographics, Face Recognition, and Object Recognition Performance

| **Participants** | **Age** | **Gender** | **CFMT** | **NOMT** |
| --- | --- | --- | --- | --- |
| 1 | 22 | F | 34 | 62 |
| 2 | 29 | F | 37 | 65 |
| 3 | 34 | F | 39 | 58 |
| 4 | 61 | M | 38 | 43 |
| 5 | 36 | X* | 35 | 63 |
| 6 | 33 | M | 36 | 58 |
| 7 | 27 | M | 38 | 68 |
| 8 | 46 | F | 34 | 59 |
| 9 | 53 | F | 35 | 52 |
| 10 | 26 | F | 42 | 60 |
| 11 | 35 | F | 43 | 72 |
| 12 | 30 | F | 41 | 60 |
| 13 | 32 | F | 40 | 64 |
| 14 | 27 | F | 44 | 66 |
| 15 | 63 | F | 37 | 51 |
| 16 | 31 | F | 37 | 42 |
| 17 | 55 | F | 33 | 38 |
| 18 | 39 | F | 33 | 47 |
| 19 | 28 | F | 42 | 71 |
| 20 | 37 | M | 33 | 64 |
| 21 | 28 | F | 39 | 68 |
| 22 | 64 | F | 43 | 51 |
| 23 | 52 | F | 38 | 54 |
| 24 | 25 | F | 44 | 62 |
| 25 | 50 | M | 44 | 52 |
| 26 | 33 | F | 39 | 45 |
| 27 | 39 | F | 41 | 56 |
| 28 | 23 | F | 32 | 57 |
| 29 | 70 | F | 36 | 70 |
| 30 | 27 | F | 42 | 68 |
| DP | 38.5 ± 13.7 | 24:5 | 38.3 ± 3.7 | 58.2 ± 9.1 |
| In-lab Control | 40.03 ± 11.7 | 19:11 | 59.77 ± 7.8 | 57.0 ± 8.3 |

*Note. CFMT: Cambridge Face Memory Test, NOMT: Novel Object Memory Test (Ziggerins). *This participant’s gender identity is nonbinary.*
