# Supplementary material for: Evidence for normal novel object recognition abilities in developmental prosopagnosia
Source: R Soc Open Sci. 2020 Sep 23;7(9):200988. doi: 10.1098/rsos.200988 (PMC7540787; doi:10.1098/rsos.200988)
Supplement: Supplementary Results [file rsos200988supp6.pdf]

CFMT and NOMT Individual-level Performance – Normative Web Data

We wanted to further investigate the individual scores on each task by using the normative web-based control sample to determine if there were dissociations present between face and object recognition. Using Crawford's (2003) criteria, 8 out of the 30 DPs (26.7%) showed a putative classical dissociation between their NOMT and CFMT scores. These individuals scored in the unimpaired range for the NOMT (< 2 SD below the web control mean) but were significantly impaired on the CFMT (> 2 SD below the web control mean), and their performance on the CFMT was significantly lower than their performance on the NOMT (Crawford, Howell, & Garthwaite, 1998).

Of the 22 classified as having an association, 13 had NOMT scores < 1 standard deviation below the web control mean and 18 had NOMT scores < 2 standard deviations below. The remaining 4/30 (13.3%) DPs reached major impairment on the NOMT, scoring > 2 SD below the mean accuracy of the web-based control group. This prevalence is comparable with the 10% (3/30; $p = 1.00$) rate of NOMT impairment found in our in-lab control group as well as the 5.5% (15/274; $p = .104$) prevalence in the web control sample. If the threshold for deficits is lowered to > 1.7 standard deviations below the mean (as used by Geskin and Behrmann), DPs show a prevalence rate of 5/30 (16.7%), failing to differ from the in-lab controls (4/30, 13.3%; $p = 1.00$) and only trending towards difference from the web sample's proportion (17/274, 6.2%; $p = .052$).

Because the web control group had a greater percentage of males than the DPs, we next divided the web control group by gender and compared individual DPs and controls to their respective gender's normative data to determine whether there were gender effects in the individual analyses. Using each gender's normative NOMT data ($M_{Male} = 62.6$, $SD_{Male} = 7.9$, $M_{Female} = 60.1$, $SD_{Female} = 7.9$) resulted in one male DP and two female DPs scoring more than two standard deviations below their respective gender's control mean on the NOMT. This proportion of impairment among the DPs (3/30, 10%) does not differ from the proportion of impairment seen in the in-lab control group (1 male, 2 females; 3/30, 10%). When comparing the web controls to the gender-separated normative data, 5/112 males (4.5%) and 7/160 females (4.4%) were more than two standard deviations below the control mean, for an overall impairment prevalence of 4.4% (12/272; three participants did not report gender). While lower than the impairment prevalence in DPs and in-lab controls, Fisher's exact test showed that the

difference does not reach significance ($p = .177$). When the impairment threshold is lowered to > 1.7 standard deviations below the gender-separated means, the prevalence rises to 4/30 DPs (1 male, 3 females; 13.3%), 4/30 in-lab controls (1 male, 3 females; 13.3%), and 19/272 web controls (8 males, 11 females; 7.0%). Fisher's exact test showed that the difference between prevalence in DPs and web controls at > 1.7 SD does not reach significance ($p = .265$).

Individual Reaction Time Analyses

We ran additional individual analyses to determine if including reaction time deficits affects the deficit prevalence. 2/30 (6.7%) DPs, 2/30 (6.7%) in-lab controls, and 2/274 (.73%) web controls had reaction times > 2 standard deviations above the in-lab control mean. If the threshold is lowered to 1.7 standard deviations below the control mean, 2/30 DPs, 3/30 in-lab controls, and 6/274 web controls reach impairment. Adding the additional reaction time impairments raises the overall impairment levels to 4/30 (13.3%) DPs, 5/30 (16.7%) in-lab controls, and 13/274 (4.7%) web controls. Neither the in-lab controls ($p > .05$, Fisher's exact test) nor the web controls ($p = .074$, Fisher's exact test) differed from the DP group in proportion.
